# Samarium-Promoted Layered La_2_NiO_4_ Perovskite for Hydrogen Production via the Auto-Thermal Reforming of Acetic Acid

**DOI:** 10.3390/ma18071508

**Published:** 2025-03-27

**Authors:** Xiaomin Hu, Lihong Huang, Ning Wang

**Affiliations:** 1 College of Environmental Science and Engineering, Beijing University of Technology, Beijing 100124, China; hxm15208@emails.bjut.edu.cn; 2 Department of Chemical and Pharmaceutical Engineering, Chengdu University of Technology, Chengdu 610059, China; 3 State Key Laboratory of Geohazard Prevention and Geoenvironment Protection, Chengdu University of Technology, Chengdu 610059, China

**Keywords:** acetic acid, auto-thermal reforming, hydrogen, La_2_NiO_4_, Sm doping

## Abstract

The auto-thermal reforming (ATR) of acetic acid is an effective hydrogen production method, but it suffers from catalyst deactivation by coking. Sm-promoted layered La_2_NiO_4_ perovskite catalysts were synthesized via the sol–gel method and its catalytic performance in the ATR of HAc was further evaluated. The characterization results demonstrate that the incorporation of Sm into the lattice of La_2_NiO_4_ perovskite led to the formation of Ni-La-Sm-O species, inducing crystal defects in the perovskite structure which could promote the gasification of coking precursors. Additionally, Sm regulated the reduction characteristics of La_2_NiO_4_, resulting in the formation of highly dispersed nickel nanoparticles upon the hydrogen reduction, which increased the number of active sites available for acetic acid conversion. Consequently, a stable reactivity without obvious coking was obtained over a Ni_0.42_La_0.7_Sm_0.36_O_2.01±δ_ catalyst within the ATR of Hac. The hydrogen yield reached 2.53 mol-H_2_/mol-HAc along with the complete conversion of acetic acid.

## 1. Introduction

The rapid advancements in industry of the 21st century have significantly propelled the development of energy systems. However, the prevailing energy framework remains heavily reliant on traditional fossil fuels, which cause irreversible environmental degradation. This situation necessitates a transition towards diversified and sustainable energy sources [1,2]. Biofuel, derived from the fermentation or pyrolysis of biomass, is a renewable biological resource, and the synthesis process does not emit additional carbon dioxide into the natural environment [3,4]. The further conversion of biofuel into environmentally friendly hydrogen has very broad application prospects in the energy field [5,6,7].

Acetic acid (HAc), which constitutes approximately 33.0 wt% of bio-oil, is regarded as a viable hydrogen source [8,9]. Hydrogen production from HAc can be achieved through conventional steam reforming (SR), as delineated in Equation (1). However, its practical industrial application was constrained by the endothermic nature of the process with a ΔH of 131.4 kJ/mol [10,11]. In contrast, by modifying the ratio of gaseous oxygen in the feed, the heat network in SR can be effectively optimized, thereby allowing the auto-thermal reforming (ATR) process to achieve energy self-sufficiency with a ΔH of 0 kJ/mol (Equation (2)) [12,13]. There have been many efforts for the industrial application of ATR with resources of fuel and kerosene [14], natural gas [15], and ethanol [16], which inspired the development of novel catalysts for hydrogen production via the ATR of HAc.

CH_3_COOH + 2H_2_O → 2CO_2_ + 4H_2_       ΔH = +131.4 kJ/mol (1)

CH_3_COOH + 1.44H_2_O + 0.28O_2_→ 2CO_2_ + 3.44H_2_ ΔH = 0 kJ/mol (2)

Within the catalysts that are applied in the ATR of HAc, nickel-based catalysts are favored for their cost-effectiveness and efficacy in breaking C–C bonds [17]. However, coking poses a significant challenge to nickel-based catalysts during this process. Although nickel effectively cleaves the C–C bond in key intermediates such as CH_3_CO and CH_3_COO, the derived CH_3_ species can continuously dehydrogenate to C species, which serve as precursors for coke deposition, ultimately leading to catalyst deactivation [18,19]. In previous nickel-based catalysts in ATR of HAc, the Ca-Al-layered double hydroxides structure [11], ordered mesoporous Al_2_O_3_ structure [12], and Cr-promoted ZnNi intermetallic compound [20] show a high reactivity in hydrogen production, but the formation of coke was observed over the spent catalysts, indicating coking was a hard to solve question of nickel-based catalysts in the ATR of HAc.

To mitigate this issue over nickel-based catalysts in the ATR of HAc, the layered perovskite-like structure of A_2_BO_4_ was selected. The perovskite structure can be preserved when the elements in an A or B site were partly substituted [21], and more importantly, the substituted perovskite can yield defect sites, which enhance the activation of H_2_O and O_2_ to produce active O species, facilitating the oxidation of carbon precursors [22]. Consequently, the coke resistance of nickel-based catalysts in the ATR of HAc was enhanced. In addition, the stable layered perovskite-like A_2_BO_4_ structure show high redox properties, which was beneficial to the dispersion of the nickel species and provides more active sites for acetic acid activation [13].

Accordingly, in current work, a Sm-doped layered perovskite-like La_2_NiO_4_ catalyst was obtained via the sol–gel method, and was evaluated in the ATR of HAc for hydrogen production. Moreover, XRD, TPR, BET, TG, and SEM were employed to carefully screen the precursors, oxides, and reduced and spent catalysts to study the evolution of the catalysts, and the relationship between the structure and catalytic performance was also discussed. The optimal Sm-doped La_2_NiO_4_ catalyst (NLS30) with more defect sites exhibited a high hydrogen yield at 2.53 mol-H_2_/mol-HAc with a HAc conversion maintained at 100%, while the coke resistance was improved as well.

## 2. Materials and Methods

### 2.1. Catalyst Preparation

The Ni-La-Sm catalysts were prepared by the sol–gel method as previously described in our work [18]. Typically, a mixture of nickel nitrate, lanthanum nitrate, and samarium nitrate (Chengdu Kelong Chemical Reagent Factory, Chengdu, China) were obtained under vigorous stirring. Then, citric acid and ethylene glycol (Chengdu Kelong Chemical Reagent Factory, Chengdu, China) was added to the above solution to be hydrated at 60 °C to form a gel, while the molar content of the citric acid and ethylene glycol was equal to the sum of the metal ions. After being dried in the oven at 105 °C for 12 h, the precursor was then calcined at 700 °C for 4 h with a temperature gradient of 10 °C/min. The as-made catalysts with different Sm loading (mass fraction = 0%, 15%, 30% and 50 wt%) were denoted as NL, NLS15, NLS30, and NLS50, while the Ni loading was always kept at 15wt%.

### 2.2. Catalytic Performance Test

**Figure 1:**
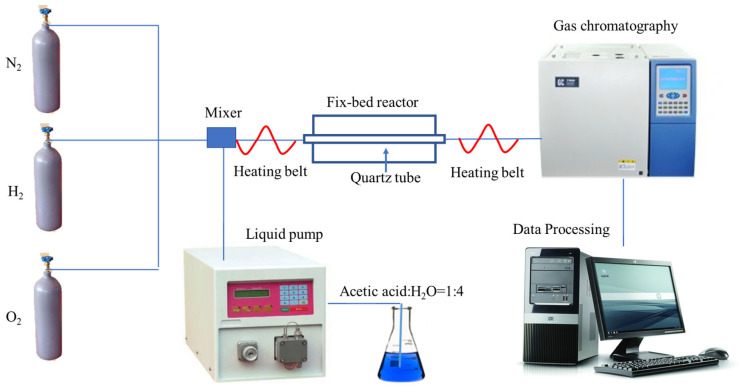
The device diagram of the auto-thermal reforming of acetic acid.

The auto-thermal reforming reaction was performed on a fix-bed quartz tubing reactor. After loading the catalyst in the middle of the reactor tube, pure H_2_ was introduced to pretreat the catalyst at 700 °C for 1 h. Then, the liquid reactant of acetic acid and water was pumped by a liquid pump (Elite, Dalian, China), which was heated to 230 °C by the heating belt. The gaseous O_2_ was then introduced into the reaction system with N_2_ as internal standard gas, while the molar ratio of HAc:H_2_O:O_2_:N_2_ was kept at 1:4:0.28:3. In addition, the gas hour space velocity (GHSV) was kept at 50,000 mL g_catal_^−1^ h^−1^. The outlet gas of the reactor was detected by the combination of a thermal conductivity detector (TCD, connected with a carbon molecular sieves column) and a flame ionization detector (FID, equipped with a Porapak Q column) within the SC-3000B gas chromatography device (Chuanyi Instrument, Chongqing, China). The device diagram is shown in Figure 1. Moreover, the selectivity of carbon-containing products (Si), the HAc conversion (X_HAc_), and the hydrogen yield (YH_2_) were calculated by Equations (3)–(5), respectively.

(3) $$S_{i\;\mathrm{carbon}-\mathrm{containing}\;\mathrm{product}\;}=\frac{F_{i\;\mathrm{carbon}-\mathrm{containing}\;\mathrm{product}}}{n_{i}(F_{\mathrm{HAc}\;\mathrm{in}}-F_{\mathrm{HAc}\;\mathrm{out}})}$$

(4) $$X_{\mathrm{HAc}}=\frac{F_{\mathrm{HAc}\;\mathrm{in}}-F_{\mathrm{HAc}\;\mathrm{out}}}{F_{\mathrm{HAc}\;\mathrm{in}}}$$

(5) $$Y_{H_{2}}=\frac{F_{H_{2}\;\mathrm{product}}}{F_{\mathrm{HAc}\;\mathrm{in}}}$$

In the above equations, Fi, in or out, represents the molar flow of i species at the inlet or at the outlet of the reactor, while ni means the stoichiometric ratio of carbon between the carbon-containing products and acetic acid.

### 2.3. Catalyst Characterizations

The crystal information of the calcined, reduced, and spent catalysts were investigated by X-ray diffraction (XRD) via the Rigaku Ultima IV X-ray diffractometer (Tokyo, Japan) with Cu-Kα radiation at 40.0 kV and 30 mA from 5° to 80°.

A JW-BK112 automatic adsorption instrument (JWGB, Beijing, China) was applied to record the nitrogen physisorption of the calcined catalysts, which operated at −196 °C. The specific surface area (SSA) was calculated by the Bruner–Emmett–Teller (BET) equation, while the pore size distribution and pore volumes were calculated by the Barrett–Joyner–Halenda (BJH) model with the relative pressure (P/P_0_) in the range from 0.7–0.98.

After being in situ preheated at 300 °C in pure nitrogen, the 50 mg catalyst was then cooled down to 50 °C and treated in a 5% H_2_/N_2_ flow. Subsequently, the H_2_-temperature programmed reduction (H_2_-TPR) was conducted by heating from 50 °C to 900 °C with a gradient of 10 °C/min and kept at 900 °C for another 30 min, while the consumption of hydrogen was continuously monitored by a TCD of a TP-5076 apparatus (Xianquan Instrument, Tianjin, China).

To probe the coke formation during the reaction, thermogravimetry (TG) and differential thermal analysis (DTA) of the spent catalysts were conducted on a SHIMADZU DTG-60 apparatus (Kyoto, Japan) in air by heating up to 800 °C with a heating ramp of 10 °C·min^−1^.

The morphologic features of the catalysts were characterized by scanning electron microscopy (SEM, Inspect F50, FEI, Hillsboro, OR, USA). The catalyst powder was sputtered with gold in a vacuum chamber prior to measurement.

## 3. Results and Discussion

### 3.1. Characterization of Calcined Catalysts

**Figure 2:**
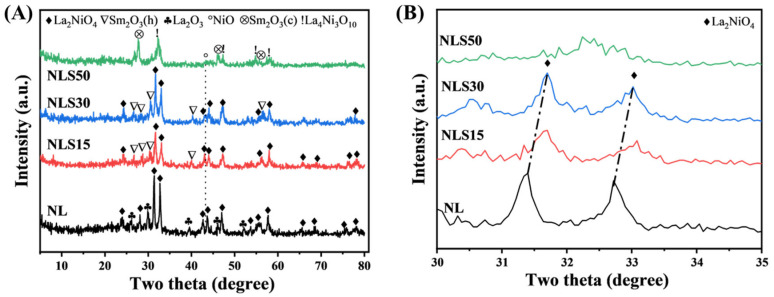
(**A**) XRD patterns of calcined NLS catalysts (Cu-Kα, 40 kV, and 30 mA, 2θ range: 10–80°) and (**B**) enlarged XRD patterns of calcined NLS catalysts from 30° to 35° (Cu-Kα, 40 kV, and 30 mA).

The crystal structure was detected by XRD to verify the formation of perovskite structure. As shown in Figure 2, for the binary LS catalyst without samarium, the characteristic peaks of a layered La_2_NiO_4_ perovskite structure appeared in the XRD spectrum. Additionally, the presence of excess La contributed to the observable La_2_O_3_ phase. Upon adding 15 wt% Sm_2_O_3_, the diffraction peaks of the layered perovskite still existed over NLS15, but shifted towards a higher degree compared to the NL catalyst (Figure 2B), indicating that the Sm component was successfully incorporated into the La_2_NiO_4_ lattice to form a composite Ni-La-Sm perovskite phase [23]. Considering the ionic radii of Sm^3+^ and La^3+^ was 0.96 Å and 1.06 Å, respectively, the incorporation of Sm into La_2_NiO_4_ perovskite to replace the site of La will cause a lattice shrink, as reflected in the peak shifting towards a higher angle. At the same time, the characteristic peaks of La_2_O_3_ disappeared, and the diffraction peaks of the hexagonal phase Sm_2_O_3_ appeared [24]. For the NLS30 catalyst, the diffraction peaks were similar to those of NLS15, but with an increased intensity. When Sm loading reached 50%, the La_2_NiO_4_ transferred to La_4_Ni_3_O_10_, indicating that the addition of excessive Sm led to the transformation of the perovskite phase [25]. In addition, the phase of Sm oxide also changed from hexagonal Sm_2_O_3_ to cubic Sm_2_O_3_. It is worth noting that the peak of NiO was only observed in the diffraction spectrum of NLS30 and NLS50, indicating that the excess Sm promoted the separation of NiO from the perovskite phase.

**Figure 3:**
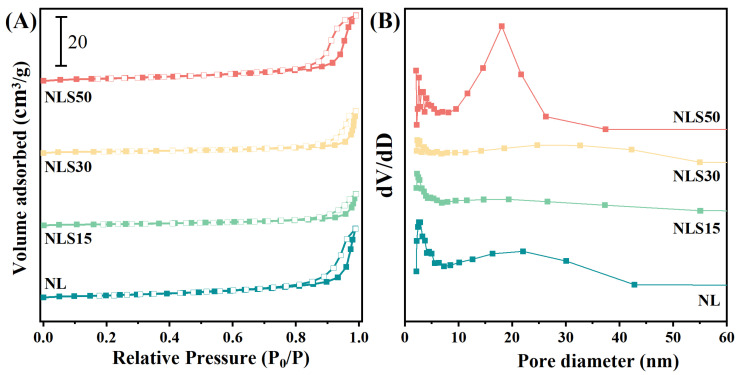
(**A**) N_2_ adsorption–desorption isotherms and (**B**) pore size distribution curves of the calcined catalysts.

In addition, nitrogen physical adsorption and desorption combined with the Bruner–Emmett–Teller (BET) method and the BJH model were used to further describe the pore structure characteristics of the oxide catalysts, as shown in Figure 3. As can be seen from Figure 3A, the isothermal adsorption and desorption curves for the four catalysts all belong to type IV, and their hysteresis loops can be classified as type H4, indicating these four catalysts were slit-type mesoporous materials [26]. It can also be intuitively seen from the isothermal adsorption and desorption curves that the NL and NLS50 catalysts have larger pore volumes and specific surface areas, and the pore size distribution of NLS50 was more concentrated with an average pore size of 17.5 nm (Figure 3B and Table 1). In contrast, the average pore size of NL is 18.1 nm. It can be seen from Table 1 that with the addition of Sm, the average pore size of NLS15 and NLS30 gradually increases, while the specific surface area gradually decreases. In actual, with the addition of Sm into NL catalysts (NLS15 and NLS30), the specific surface area of the NL catalysts was gradually decreased along with a decreased pore volume, which could be reasonably attributed the structural change of La_2_NiO_4_ perovskite and possible pore collapse. However, with Sm additionally further reaching 50 wt% (NLS50), the main phases were La_4_Ni_3_O_10_ and hexagonal Sm_2_O_3_ rather than Sm-doped La_2_NiO_4_ perovskite, and the corresponding phase transition resulted in the enhanced surface area and pore volume compared to NLS30 and NLS15. More specifically, the pore size distribution was definitely an important factor that correlates with catalytic activity in some catalytic systems. However, in this work, as shown in Table 1, the average pore size was similar for NL, NLS15, and NLS50 (18.1 nm for NL, 17.6 nm for NLS15, and 17.5 nm for NLS50), but there is a huge difference in the reactivity. Therefore, the pore size distribution has little effect on the reactivity, and what really determines the reactivity was the highly dispersed nickel nanoparticles and lattice defects as will be discussed in the following content.

### 3.2. Characterization of Reduced Catalysts

**Figure 4:**
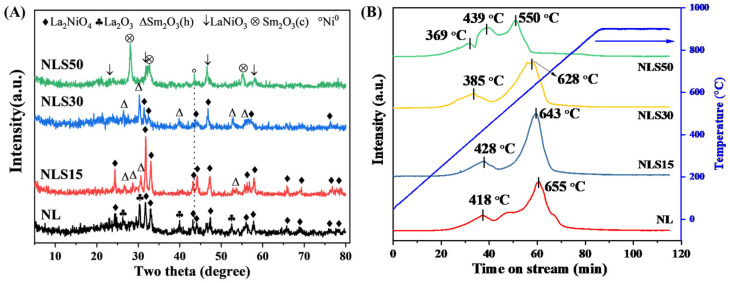
(**A**) XRD patterns of reduced NLS catalysts (Cu-Kα, 40 kV, and 30 mA, 2θ range: 10–80°) and (**B**) H_2_-TPR profiles of calcined catalysts (the right Y-axis represents temperature).

After being reduced at 700 °C in hydrogen for 1 h, the crystal structure was also determined by XRD. As displayed in Figure 4, for the reduced NL catalyst, the layered La_2_NiO_4_ perovskite and La_2_O_3_ still exist stably, but the peak intensity of La_2_NiO_4_ was weakened, indicating that part of La_2_NiO_4_ was reduced by hydrogen. Over the NLS15 catalyst, the diffraction peaks of La_2_NiO_4_ and hexagonal Sm_2_O_3_ were observed, which was consistent with the phases that existed in the calcined catalysts. With the increase of Sm loading, the peak intensity of Sm_2_O_3_ over the reduced NLS30 catalyst was increased, while the peak intensity of La_2_NiO_4_ was weakened. While adding excessive Sm, the Sm-containing phase in NLS50 was transformed into cubic Sm_2_O_3_. At the same time, the peaks that belonged to La_4_Ni_3_O_10_ disappeared with the emergence of LaNiO_3_ perovskite [27]. As for the active metallic nickel, only a strong diffraction peak was detected in NLS50, while no related peaks were found in the other three catalysts. The reason behind this may connect with the high nickel dispersion via the reduction of perovskite and is therefore not detected by XRD [28].

To further investigate the evolution of nickel species during reduction, the calcined NLS catalysts were subjected to a H_2_-TPR test. As shown in Figure 4B, two peaks appeared in the NL, of which the peak at 418 °C corresponds to the reduction of bulk NiO, while the peak at 655 °C was attributed to the reduction of Ni species in the perovskite La_2_NiO_4_ phase. With the addition of Sm, the reduction temperature of Ni species in the perovskite phase over NLS15 and NLS30 gradually decreased. The reduction temperature of Ni in Sm-doped La_2_NiO_4_ over NLS30 decreased to 628 °C, indicating that the introduction of Sm regulated the interaction between Ni and the perovskite phase, and further promoted the reduction of Ni in the perovskite. For the NLS50 catalyst, the reduction temperature of bulk NiO was significantly reduced to 369 °C. In addition, the reduction peak at 550 °C corresponds to the reduction in La_4_Ni_3_O_10_, and the reduction peak at 439 °C belongs to the reduction of the Ni-containing phase in LaNiO_3_ which is derived from the reduction of La_4_Ni_3_O_10_. However, the hydrogen consumption over NLS50 was smaller than the other catalysts, indicating the lower reduction degree of Ni species.

### 3.3. Catalytic Performance of the NLS Catalyst

#### 3.3.1. Reactivity in ATR of HAc

**Figure 5:**
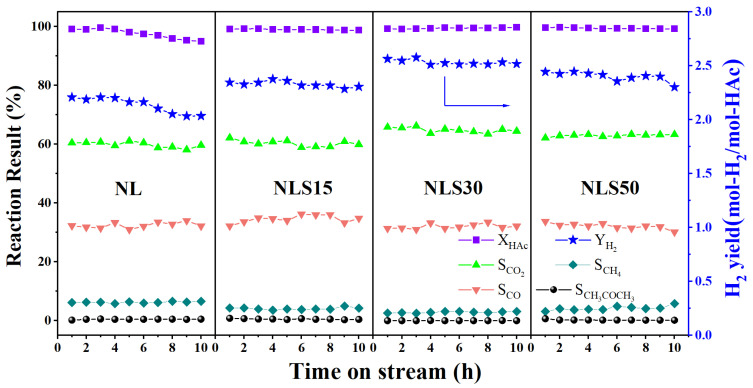
The catalytic performance of NLS catalysts at 650 °C, atmosphere pressure, and GHSV = 50,000 mL·g_catal_^−1^ h^−1^ for the ATR of HAc.

After being reduced at 700 °C in hydrogen for 1 h, the catalyst was further evaluated for hydrogen production in an auto-thermal reforming of acetic acid under 650 °C, atmospheric pressure, and GHSV = 50,000 mL·g_catal_^−1^ h^−1^. As shown in Figure 5, in the first 4 h, the conversion rate of acetic acid was close to 100% for the NL catalyst, but the hydrogen yield was as low as 2.20 mol-H_2_/mol-HAc. During the reaction time of 5–10 h, the HAc conversion and the hydrogen yield gradually decreased, and finally reached 94.9% and 2.03 mol-H_2_/mol-HAc, respectively. In addition, the selectivity of carbon-containing products of CO and CO_2_ fluctuated around 61% and 32%, respectively. Although acetone was presented in a low concentration, methane exhibited a relatively high selectivity (~6%), indicating significant methanation activity for the NL catalyst, which contributed to the decreased hydrogen yield.

With the incorporation of Sm, the catalytic performance of NLS15 was improved and the acetic acid conversion remained at 100%. Although the selectivity of carbon monoxide and carbon dioxide was similar to that of the NL catalyst, the selectivity of methane dropped to 4%, and, therefore, the hydrogen yield increased and stabilized at 2.31 mol-H_2_/mol-HAc within a period of 10 h.

While the loading of Sm reached 30wt%, the NLS30 catalyst exhibited excellent catalytic activity and stability in the ATR of HAc, with an acetic acid conversion close to 100% and a hydrogen yield stable at 2.53 mol-H_2_/mol-HAc. Due to the influence of the water–gas shift reaction (CO + H_2_O → CO_2_ + H_2_), the selectivity of CO_2_ over the NLS30 catalyst increased to 64%, while the selectivity of CO decreased to 31%, along with the hydrogen yield higher than the other three catalysts [29]. In addition, the selectivity of methane over the NLS30 catalyst was as low as 3%, and the presence of acetone is basically not detected.

The NLS50 catalyst also exhibited a comparable activation ability for acetic acid, with HAc conversion recording near 100% within 10 h, but the hydrogen yield decreased from 2.44 mol-H_2_/mol-HAc to 2.30 mol-H_2_/mol-HAc. Additionally, the selectivity of methane also increased from 3% to 6%, indicating its high methanation reactivity [30]. The above reactivity results indicated that the activity and stability of the NLS50 catalyst decreased with excess Sm loading.

#### 3.3.2. The Reactivity of NLS Catalysts Under Different Temperatures

**Figure 6:**
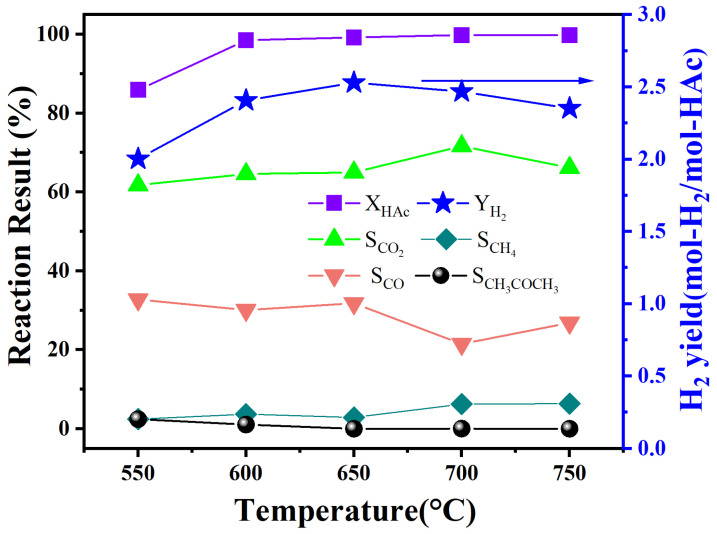
The effect of temperature on the catalytic performance of the NLS30 catalyst in the ATR of HAc at GHSV = 50,000 mL·g_catal_^−1^ h^−1^.

From the results in Figure 5, it can be seen that the NLS30 catalyst with an appropriate amount of Sm showed a relatively higher catalytic activity and stability in the auto-thermal reforming of acetic acid. Therefore, this catalyst was selected as the research object to further explore the effect of reaction temperature on the catalytic activity. The corresponding results were shown in Figure 6, while the reaction condition was atmospheric pressure and GHSV = 50,000 mL·g_catal_^−1^ h^−1^, and the selected temperature range was from 550 °C to 750 °C with 50 °C as the separation interval.

As shown in Figure 6, at a low temperature of 550 °C, the acetic acid conversion was only 86%, and the hydrogen yield is 2.00 mol-H_2_/mol-HAc. In addition, the selectivity of the acetone reached 2.4%, indicating that the acetonization reaction was more favorable at a lower temperature (CH_3_CO + CH_3_ → CH_3_COCH_3_) [31]. The selectivity of CO and CO_2_ at this temperature was about 61% and 32%, respectively. In the temperature range of 550 –650 °C, the increase in temperature intensifies the water–gas shift reaction, so the hydrogen yield also increases with the increase of temperature, reaching 2.53 mol-H_2_/mol-HAc at 650 °C. On the other hand, the increased temperature further promoted the thermodynamic decomposition of acetic acid was nearly completely converted at 650 °C. However, as the temperature further increases, the selectivity of acetone gradually decreases and the selectivity of methane increases, indicating that for NLS30, a high temperature inhibits the acetonization reaction of acetic acid but intensifies the methanation reaction. Therefore, at higher reaction temperatures (700 °C and 750 °C), part of the hydrogen atoms in the reactant were converted into methane, resulting in a lower hydrogen yield. In summary, 650 °C was suitable for selecting the reaction temperature of the NLS30 catalyst.

### 3.4. Characterizations of Spent Catalysts

**Figure 7:**
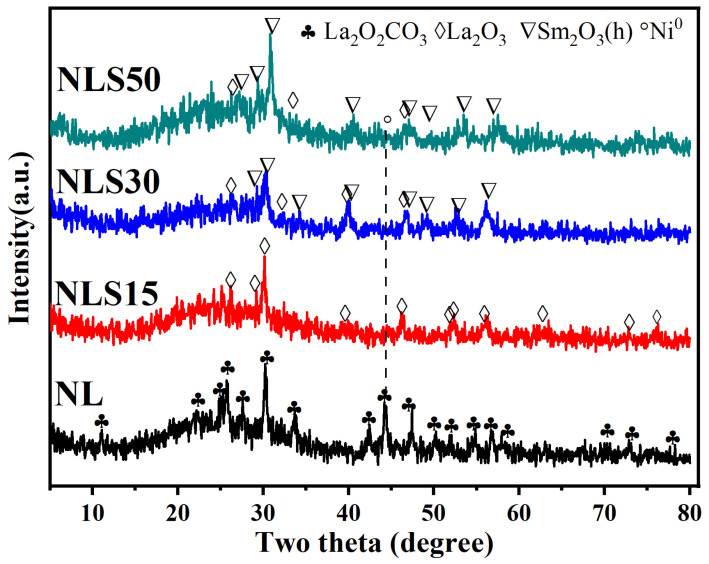
XRD patterns of spent NLS catalysts (Cu-Kα, 40 kV, and 30 mA, 2θ range: 10–80°).

The phases presented in the spent catalyst were detected by X-ray, as shown in Figure 7. For the binary NL catalyst, the perovskite structure disappeared, and obvious diffraction peaks of La_2_O_2_CO_3_ appeared, proving that La_2_O_3_ and CO_2_ combined during the reaction [32]. In addition, a strong diffraction peak of metallic nickel appeared over the NL catalyst with a particle size of 32.5 nm. In contrast, only strong diffraction peaks of La_2_O_3_ appeared in the spent NLS15 catalyst. For the spent NLS30 catalyst, the La_2_NiO_4_ phase disappeared, but the diffraction peaks of La_2_O_3_ and hexagonal Sm_2_O_3_ appeared, indicating La_2_NiO_4_ decomposed into La_2_O_3_ in the oxidizing atmosphere within the auto-thermal reforming of acetic acid. Furthermore, no peak of metallic nickel was detected over the spent NLS30 catalyst, which may be caused by the high dispersion of metallic nickel. Compared with the XRD spectrum after reduction, it can be seen that the LaNiO_3_ phase in NLS50 was transformed into La_2_O_3_ after the reaction. At the same time, the cubic Sm_2_O_3_ was transformed into the hexagonal Sm_2_O_3_ after the reaction, indicating that the hexagonal Sm_2_O_3_ has good stability in the oxidizing atmosphere.

**Figure 8:**
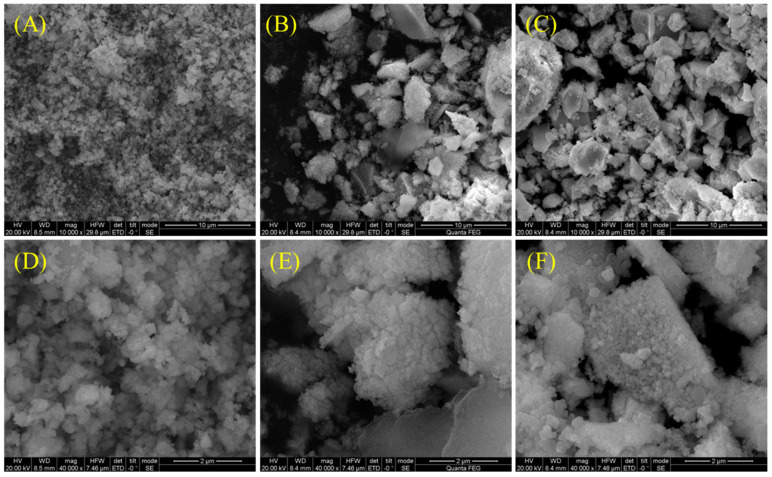
SEM images of the spent catalysts: (**A**,**D**) NL, (**B**,**E**) NLS30, and (**C**,**F**) NLS50.

**Figure 9:**
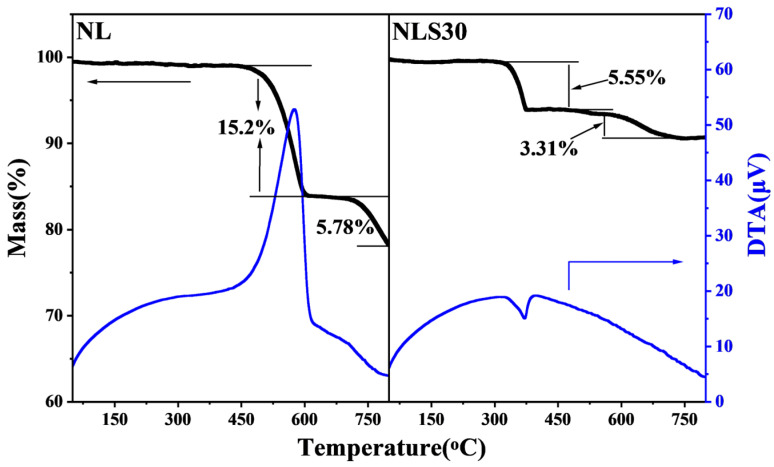
The TG-DTG profile of spent NLS catalysts.

The morphology of the spent catalyst was also characterized, and the results are shown in Figure 8. The structure of the NL catalyst after the reaction was dense, and the particles were densely packed. However, the particle size of the spent NLS30 and NLS5 catalysts was larger with more voids. The possible carbon deposition of the spent catalyst was analyzed by TG-DTG, as shown in Figure 9. For the NL catalyst without the Sm addition, the weight loss peak at 400 °C was attributed to the decomposition peak of La_2_O_2_CO_3_ and the combustion peak of filamentous carbon, while the total weight loss at this temperature reached 15.2% [13]. In contrast, the peak that connected with the combustion of graphite carbon was accompanied by a weight loss percentage of 5.78% above 650 °C [33]. The weight loss peak at 300 °C over the spent NLS30 catalyst was classified as the decomposition of La(OH)_3_, which may be generated by the absorption of water over La_2_O_3_. The subsequent weight loss peak was attributed to the combustion of graphite carbon, and its weight loss percentage was 3.31%, indicating that the carbon deposition over NLS30 was suppressed after the addition of the Sm component.

## 4. Discussion

Based on the above characterization, it can be seen that the NL catalyst presented La_2_NiO_4_ as the main phase, with the co-existence of the La_2_O_3_ phase. The results of nitrogen physical adsorption and desorption showed that the pore structure of the catalyst was mainly mesoporous, with a specific surface area and pore volume of 4.67 m^2^/g and 0.048 cm^3^/g, respectively. After the reduction with hydrogen at 700 °C for 1 h, the layered La_2_NiO_4_ perovskite and La_2_O_3_ still existed. It can also be seen from the H_2_-TRR spectrum that the reduction temperature of the nickel in La_2_NiO_4_ was high, indicating the strong interaction between nickel and the carrier. When it was evaluated in the ATR of HAc for hydrogen production, the acetic acid conversion and the hydrogen yield gradually decreased with the progress of the reaction, reaching 94.9% and 2.03 mol-H_2_/mol-HAc at the end of the 10-h testing. At the same time, a high selectivity of methane (6%) was recorded during the reaction, which may be attributed to the large nickel particle as revealed in the XRD of the spent NL catalysts. Furthermore, the TG-TDA profile showed that there was a lot of carbon deposits over the spent NL catalysts.

For NLS15 and NLS30 catalysts, the BET results indicated that with the addition of Sm, the specific surface area and pore volume all decreased. According to the XRD spectrum, it can be found that the main phase was still La_2_NiO_4_, and the hexagonal phase Sm_2_O_3_ appeared. In addition, the diffraction peak of the La_2_NiO_4_ shift towards a higher angle with the increase of Sm, indicating Sm was successfully incorporated into the lattice of La_2_NiO_4_, caused lattice defects in the perovskite structure. With Sm species successfully entering the perovskite structure, the generation of surface defects and lattice defects was promoted. Therefore, more oxygen-containing reactant molecules, such as H_2_O and O_2_, can be adsorbed and activated at the surface defect sites to generate oxygen-containing intermediates such as OH and O. The OH and O species could further oxidize the CH_3_ and CH_x_ (x = 0–2) intermediates, which are derived from the decomposition of CH_3_CO(O) species at the Ni site (CH_3_CO(O) → CH_3_ + CO/CO_2_), thereby inhibiting the formation of carbon deposits and improving the stability of the catalyst. The H_2_-TPR results also showed that the addition of Sm modulated the interaction between Ni and the carrier, which decreased the reduction temperature of Ni species in La_2_NiO_4_. With a hydrogen pretreatment, the perovskite La_2_NiO_4_ and Sm_2_O_3_ existed stably, while metallic nickel was not detected by XRD due to its high dispersion and the highly dispersed nickel species may account for the higher reactivity. Consequently, the NLS30 catalyst exhibited good activity and stability in the 10 h reaction. The acetic acid molecules were completely converted while the hydrogen production was stable at 2.53 mol-H_2_/mol-HAc.

As the Sm doping amount continues to increase to 50%, the Sm_2_O_3_ achieved phase transformation from hexagonal to cubic, resulting in the increased specific surface area and pore volume compared to NLS30, reaching 4.78 m^2^/g and 0.046 cm^3^/g, respectively. At the same time, the main phase was La_4_NiO_10_ rather than La_2_NiO_4_, which exhibited a weaker metal support interaction than La_2_NiO_4_. After reduction, the cubic Sm_2_O_3_ was retained, while the La_4_NiO_10_ phase transformed into the LaNiO_3_ perovskite. Due to the weak interaction between Ni and the carrier, the nickel species was easier to be reduced in hydrogen and the result of XRD showed an obvious diffraction peak of metallic nickel with a larger particle size, which may connect with a higher methane selectivity. In addition, the cubic Sm_2_O_3_ was not stable in this condition, which transformed into the hexagonal Sm_2_O_3_ after 10 h of reaction. Accordingly, the reactivity of the NLS50 catalyst was slightly decreased. The hydrogen yield over the NLS50 catalyst decreased from 2.44 mol-H_2_/mol-HAc to 2.30 mol-H_2_/mol-HAc, and the selectivity of methane increased from 3% to 6%.

## 5. Conclusions

In this work, a Ni-La-Sm perovskite catalyst was prepared by the sol–gel method. It can be seen that the main phase was La_2_NiO_4_ over the NL catalyst. With the incorporation of Sm, the diffraction peaks of La_2_NiO_4_ shift towards a higher angle, indicating the formation of Sm-doped La-Ni perovskite. The characterization results showed that the binary NL catalyst displayed a poor activity and more carbon deposits were formed after the reaction. In contrast, with the doping of Sm, more surface defects and structural defects were generated in the perovskite structure, which could more effectively promote the transfer activation of O and OH species and the gasification reaction of CH_3_ and CH_x_ (x = 0–2), so the carbon deposition was inhibited. At the same time, the Ni dispersion was higher over the Sm-doped La_2_NiO_4_ perovskite after hydrogen reduction, which increased the contact area between Ni species and the carrier, provided more active sites, and showed a relatively excellent catalytic activity. Consequently, the NLS30 catalyst achieved a stable hydrogen yield at 2.53 mol-H_2_/mol-HAc with a higher coke resistance. However, the introduction of excessive Sm will lead to the formation of the La_4_Ni_3_O_10_ phase, which led to the formation of large nickel particles along with a decreased hydrogen yield. Nevertheless, the quantitative analysis of active nickel species was lacking, which can be explored in further work.

## Figures and Tables

**Table 1:** N_2_ adsorption–desorption tests of NLS catalysts.

Catalysts	Specific Surface Area (m^2^/g)	Pore Volume (cm^3^/g)	Average Pore Size (nm)
NL	4.67	0.048	18.1
NLS15	2.34	0.022	17.6
NLS30	2.62	0.028	25.9
NLS50	4.78	0.046	17.5

## Data Availability

The original contributions presented in this study are included in the article. Further inquiries can be directed to the corresponding authors.
